# Proton Pump Inhibitors Decrease Eotaxin-3 Expression in the Proximal Esophagus of Children with Esophageal Eosinophilia

**DOI:** 10.1371/journal.pone.0101391

**Published:** 2014-07-02

**Authors:** Jason Y. Park, Xi Zhang, Nathalie Nguyen, Rhonda F. Souza, Stuart J. Spechler, Edaire Cheng

**Affiliations:** 1 Esophageal Diseases Center, Department of Pathology, Children's Medical Center, Eugene McDermott Center for Human Growth and Development, and the University of Texas Southwestern Medical Center, Dallas, Texas, United States of America; 2 Esophageal Diseases Center, Department of Internal Medicine, VA North Texas Health Care System, and the University of Texas Southwestern Medical Center, Dallas, Texas, United States of America; 3 Department of Pediatrics, Children's Medical Center, and the University of Texas Southwestern Medical Center, Dallas, Texas, United States of America; 4 Esophageal Diseases Center, Department of Internal Medicine, VA North Texas Health Care System, Harold C. Simmons Comprehensive Cancer Center, and the University of Texas Southwestern Medical Center, Dallas, Texas, United States of America; Vanderbilt University, United States of America

## Abstract

**Objective:**

Besides reducing gastric acid secretion, proton pump inhibitors (PPIs) suppress Th2-cytokine-stimulated expression of an eosinophil chemoattractant (eotaxin-3) by esophageal epithelial cells through acid-independent, anti-inflammatory mechanisms. To explore acid-inhibitory and acid-independent, anti-inflammatory PPI effects in reducing esophageal eosinophilia, we studied eotaxin-3 expression by the proximal and distal esophagus of children with esophageal eosinophilia before and after PPI therapy. *In vitro*, we studied acid and bile salt effects on IL-13-stimulated eotaxin-3 expression by esophageal epithelial cells.

**Design:**

Among 264 children with esophageal eosinophilia seen at a tertiary pediatric hospital from 2008 through 2012, we identified 10 with esophageal biopsies before and after PPI treatment alone. We correlated epithelial cell eotaxin-3 immunostaining with eosinophil numbers in those biopsies. *In vitro*, we measured eotaxin-3 protein secretion by esophageal squamous cells stimulated with IL-13 and exposed to acid and/or bile salt media, with or without omeprazole.

**Results:**

There was strong correlation between peak eosinophil numbers and peak eotaxin-3-positive epithelial cell numbers in esophageal biopsies. Eotaxin-3 expression decreased significantly with PPIs only in the *proximal* esophagus. In esophageal cells, exposure to acid-bile salt medium significantly suppressed IL-13-induced eotaxin-3 secretion; omeprazole added to the acid-bile salt medium further suppressed that eotaxin-3 secretion, but not as profoundly as at pH-neutral conditions.

**Conclusion:**

In children with esophageal eosinophilia, PPIs significantly decrease eotaxin-3 expression in the *proximal* but not the distal esophagus. In esophageal squamous cells, acid and bile salts decrease Th2 cytokine-stimulated eotaxin-3 secretion profoundly, possibly explaining the disparate PPI effects on the proximal and distal esophagus. In the distal esophagus, where acid reflux is greatest, a PPI-induced reduction in acid reflux (an effect that could increase eotaxin-3 secretion induced by Th2 cytokines) might mask the acid-independent, anti-inflammatory PPI effect of decreasing cytokine-stimulated eotaxin-3 secretion.

## Introduction

In eosinophilic esophagitis (EoE), food allergens trigger a T-helper 2 (Th2) immune response with production of Th2 cytokines such as interleukin (IL)-13 and IL-4 [1], [2]. These cytokines can stimulate the esophagus to express eotaxin-3, a potent eosinophil chemoattractant thought to play a key role in causing esophageal eosinophilia in EoE [3]–[5]. Esophageal eosinophilia underlies the esophageal dysfunction and tissue remodeling responsible for dysphagia and other symptoms that can seriously impair quality of life for EoE patients [6]–[8]. Consequently, a major goal of EoE treatment is to reduce esophageal eosinophil numbers [6].

Esophageal eosinophilia also can be a manifestation of gastroesophageal reflux disease (GERD), which can cause symptoms and endoscopic abnormalities similar to those of EoE [9]. To distinguish these two disorders in patients with esophageal eosinophilia, authorities have recommended a trial of proton pump inhibitor (PPI) therapy with the assumption that gastric acid inhibition is the only important effect of PPIs and, therefore, only an acid-peptic disorder like GERD can respond to PPIs. However, a number of recent observations have challenged this assumption.

PPIs have been found to have anti-inflammatory actions unrelated to their inhibitory effects on gastric acid secretion [10]. For example, PPIs inhibit cytokine production by human endothelial and tracheal epithelial cells [11], [12]. In esophageal epithelial cells in culture, we have reported that PPIs block the secretion of IL-8 and the secretion of eotaxin-3 stimulated by Th2 cytokines [5], [13], [14]. These acid-independent, anti-inflammatory effects of PPIs conceivably could contribute to resolution of esophageal eosinophilia in both GERD and EoE.

Another serious challenge to the assumption that PPI responsiveness distinguishes GERD from EoE is the recent identification of patients with PPI-responsive esophageal eosinophilia (PPI-REE). These patients have typical EoE symptoms and esophageal eosinophilia, both of which improve with PPIs even though they have no evidence of GERD by endoscopy or esophageal pH monitoring. It is not clear whether these patients respond to the acid-inhibitory effects of PPIs because they have occult GERD not detected by endoscopy and pH monitoring, or whether they respond to acid-independent, anti-inflammatory effects of PPIs because they have an immune/antigen-mediated esophageal disease (EoE or some EoE-like disorder).

The purpose of this study was to explore the contributions of acid-inhibitory and acid-independent, anti-inflammatory effects of PPIs on esophageal eosinophilia in children. We reasoned that, if PPIs reduce esophageal eosinophilia by reducing acid reflux, then those acid-inhibitory effects should manifest most prominently in the distal esophagus where acid reflux exposure is greatest. On the other hand, if PPIs reduce esophageal eosinophilia through acid-independent, anti-inflammatory effects on eotaxin-3, then those effects should manifest more equally throughout the esophagus. Therefore, we studied eotaxin-3 expression by esophageal epithelial cells in biopsy specimens of the proximal, mid, and distal esophagus of children with esophageal eosinophilia before and after PPI therapy, and correlated those findings with the number of intraepithelial eosinophils in those same biopsy specimens. *In vitro*, we studied acid and bile salt effects on Th2-cytokine-stimulated eotaxin-3 expression by esophageal epithelial cells, with or without omeprazole treatment.

## Materials and Methods

### Ethics Statement

This study was approved by the University of Texas Southwestern Medical Center Institutional Review Board (STU 032013-037). Patient clinical medical records and patient archived specimens used in this study were anonymized and de-identified prior to analysis, and the need for informed consent was waived.

### Subjects

This study was approved by the University of Texas Southwestern Medical Center Institutional Review Board (STU 032013-037). We reviewed the electronic medical records of patients evaluated in the Pediatric Gastroenterology, Hepatology, and Nutrition program at Children's Medical Center between 2008 and 2012 to identify children who had an endoscopy with esophageal biopsies showing ≥15 eosinophils per high power field (eos/hpf) and who had follow-up endoscopy with esophageal biopsies after treatment with PPIs alone for ≥8 weeks. Patients were excluded if, prior to the index endoscopy showing esophageal eosinophilia, they had received treatment with PPIs, swallowed topical steroids, systemic steroids, elimination diet, or elemental diet. Patients also were excluded if they received any concurrent treatment for EoE in addition to PPIs between the index and follow-up endoscopy. We recorded pertinent clinical data including demographics, presenting symptoms, coexisting atopic disease, and endoscopic findings. Cases were classified as PPI responders if the post-treatment biopsies had a peak eosinophil count <15 eos/hpf, and as PPI non-responders if the post-treatment biopsies had a peak eosinophil count ≥15 eos/hpf.

### Tissue Specimens

Archived, formalin-fixed, paraffin-embedded (FFPE) tissue blocks of patient biopsy specimens were obtained from the Department of Pathology at Children's Medical Center.

### Histopathology

Hematoxylin and eosin (H&E) stained slides were reviewed. For biopsy specimens taken at each level of the esophagus (proximal, mid, and distal), peak eosinophil counts in the squamous epithelium were determined by counting the number of eosinophils in the high power field with the greatest density of eosinophils. A BX41 microscope with an UPlanFL N 40x/0.75 objective lens with a FN22 eyepiece was used (Olympus America, Center Valley, PA); the calculated area per hpf is 0.237 mm^2^. Histopathological findings such as basal cell hyperplasia (>25% of thickness of epithelium), spongiosis, elongation of rete papillae (>two-thirds the thickness of the epithelium), and eosinophilic microabscess (≥3 eosinophils clustered at the luminal surface or within a maximum distance of one squamous cell from the luminal surface) were recorded. Subepithelial fibrosis was evaluated in biopsy specimens with sufficient lamina propria for meaningful analysis.

### Immunohistochemistry

Eotaxin-3 polyclonal goat anti-human IgG (Human CCL26/Eotaxin-3 Affinity Purified Polyclonal Ab, Catalog Number AF653, R&D systems, Minneapolis, MN) was used for immunohistochemistry. This eotaxin-3 polyclonal antibody was raised against a peptide from amino acid residues 24 (Threonine) through 94 (Leucine) of eotaxin-3. FFPE tissues were sectioned at 4 µm and mounted onto charged microscope slides. The slides were baked at 60°C for 30 minutes and then stained on a Discovery XT automated immunohistochemistry platform (Ventana Medical Systems, Tucson, AZ). The eotaxin-3 antibody was used at a final concentration of 2 µg/ml. The secondary antibody was UltraMap anti-goat horseradish peroxidase used at a prediluted concentration as a chromogenic reporter molecule (Catalog Number 760-4648, Ventana). Negative controls included the replacement of eotaxin-3 antibody with either non-specific goat IgG or saline.

### Quantification of Eotaxin-3 Immunohistochemistry

Immunostaining for eotaxin-3 was evaluated by a gastrointestinal pathologist (JYP). The peak number of eotaxin-3-positive squamous epithelial cells per hpf (400x) was counted in the area of greatest staining intensity. An eotaxin-3 positive squamous cell was identified by the presence of granular, perinuclear immunostaining. Eotaxin-3-positive eosinophils (identified by their distinctive morphological features) were not counted.

### Culture of Esophageal Squamous Cells

We used two non-neoplastic, telomerase-immortalized, esophageal squamous cell lines (EoE1-T and EoE2-T) that were created from esophageal mucosal biopsy specimens from patients with EoE as previously described by our laboratory [5]. Briefly, the patients fulfilled the criteria for EoE suggested in the 2007 consensus recommendations [15]. They both had a history of dysphagia and heartburn that had responded only partially or not at all to PPIs, and had esophageal biopsy specimens showing ≥15 eos/hpf; symptoms subsequently improved dramatically with fluticasone treatment. We also used two previously characterized, telomerase-immortalized, non-neoplastic esophageal squamous cell lines established from patients with GERD [16]. One line (NES-B10T) was created from endoscopic biopsy specimens of squamous epithelium in the esophagus of a patient who had GERD associated with long-segment Barrett's esophagus (>3 cm of specialized intestinal metaplasia). The other line (NES-G4T) was established from esophageal biopsy specimens from a GERD patient who had Los Angeles grade C reflux esophagitis without Barrett's esophagus.

Cells were maintained in monolayer culture at 37°C in humidified air with 5% CO_2_ in growth medium co-cultured with a fibroblast feeder layer as previously described [16]. For individual experiments, cells were equally seeded into collagen IV-coated wells (BD Biosciences, San Jose, CA) and maintained in growth medium.

### Cytokine Stimulation and Omeprazole Treatment of Esophageal Squamous Cells

Cells were stimulated with IL-13 (R&D Systems) in concentrations of 1 or 100 ng/ml for 48 hours. For studies involving PPI effects, omeprazole (Sigma-Aldrich, St. Louis, MO) 5 µM was acid-activated in medium with pH 5.5 for 30 minutes [5], [13], [17]. Cells were then pre-treated for 2 hours with omeprazole in medium with pH 7.2 prior to the addition of IL-13. Omeprazole remained in the media throughout the period of cytokine stimulation.

### Acid and Bile Salt Exposure of Esophageal Squamous Cells

Cells were cultured either in neutral medium (pH 7.2) alone or in neutral medium with periodic exposures to acidic media (pH levels ranging from 4.0 to 7.2) with and without bile salts. Stable cell number and viability were verified by cell counting for each of these experimental conditions. Cells exposed to pH 3.0 were not viable. The bile salt media contained a mixture of conjugated bile acids (glycocholic acid, glycodeoxycholic acid [both from Calbiochem, San Diego, CA], taurocholic acid, glycochenodeoxycholic acid, taurochenodeoxycholic acid, and taurodeoxycholic acid [Sigma-Aldrich] in a 20∶6∶3∶15∶3∶1 molar concentration, total concentration 50 µM) designed to simulate the bile acid composition of gastric refluxate described in patients with GERD and Barrett's esophagus [18]–[20]. Cells were exposed to acid and/or bile salt media for 10 minutes, three times a day, for 2 days. These exposure durations were chosen to simulate typical episodes of gastroesophageal reflux [20]. For experiments evaluating IL-13 and omeprazole effects, the media that was replaced (after the 10-minute acid and bile salt media exposure) contained IL-13 with or without omeprazole.

### Enzyme-Linked Immunosorbent Assays (ELISA) for Eotaxin-3

We performed ELISA on conditioned media after 48 hours to assess the production of eotaxin-3 by esophageal epithelial cells. Conditioned media from the cells were collected and centrifuged to remove cellular debris. Eotaxin-3 concentrations were determined using commercially-available ELISA kits (R&D Systems) per manufacturer's instructions. The absorbance of each well was read at 450 nm and 540 nm using a DTX 880 Multimode plate reader (Beckman Coulter). Results were expressed as pg/ml of eotaxin-3. All assays were performed in duplicate.

### Statistical analysis

Categorical data are expressed as frequency and/or percentage, and bivariate analysis was performed with Fischer's exact test. Continuous data are expressed as mean ± standard error of the mean (SEM), and bivariate analysis was evaluated with t-tests. Paired t-tests were used to evaluate before and after therapy results. Correlations between eosinophil counts and eotaxin-3-positive-staining epithelial cell counts were calculated by Pearson correlation coefficient ρ (R). For *in vitro* studies, data are expressed as mean ± SEM. Multivariate analysis was performed with one-way ANOVA. Statistical significance was determined by *P* value ≤0.05. Statistical analyses were performed with GraphPad Prism 6 (GraphPad Software, Inc, La Jolla, CA).

## Results

### Baseline Patient Characteristic and Clinicopathological Features

**Figure 1:**
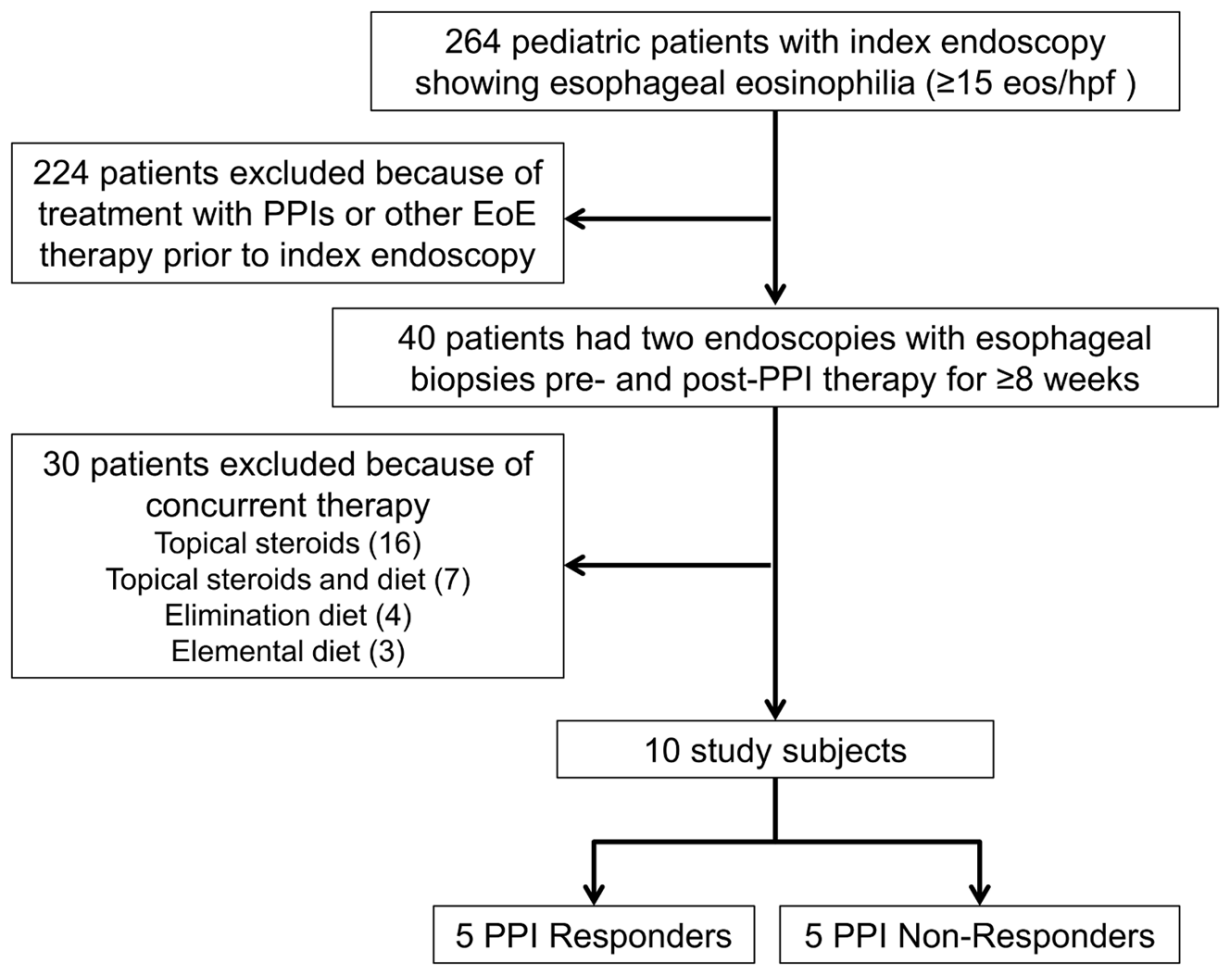
Flow diagram of study subject selection.

We identified 264 patients with esophageal eosinophilia (Figure 1). Forty patients who had been treated with PPIs had pre- and post-treatment endoscopic examinations; 30 of those 40 were excluded because they had received concurrent treatment in addition to PPIs, leaving 10 study subjects for evaluation.

Histological review of the post-treatment biopsy specimens identified 5 PPI responders (<15 eos/hpf) and 5 PPI non-responders (≥15 eos/hpf) (Table 1). There were no significant differences in baseline characteristics between the groups except for the symptoms of weight loss/poor weight gain and vomiting, which were more frequent in the PPI responders, and baseline peak eosinophil counts, which were lower in the PPI responders.

**Table 1:** Baseline Patient Characteristics.

		PPI Responders (N = 5)	PPI Non-Responders (N = 5)	*P* value*
**Demographics**	Mean age (y ± SEM)	11±2	11±2	0.97
	Male	3	5	0.44
	Caucasian	5	5	1
**Coexisting allergies**	Any atopic disease	2	3	1
	Allergic rhinitis	0	2	0.44
	Asthma	1	1	1
	Eczema	1	0	1
	Food allergies	0	1	1
**Symptoms**	Dysphagia	1	4	0.21
	Oral aversion	1	0	1
	Abdominal pain	2	1	1
	Weight loss/poor weight gain	4	0	0.047
	Food impaction	0	1	1
	Vomiting	4	0	0.047
**Endoscopic Findings**	Furrows	3	5	0.44
	Rings	0	3	0.17
	Stricture	0	1	1
	White plaques	4	1	0.21
	Edema	1	4	0.21
**Histology**	Peak eosinophils (eos/hpf ± SEM)	33.2±7.5	67.2±28.8	0.03
	Basal hyperplasia	5	5	1
	Spongiosis	5	5	1
	Papillary elongation	5	5	1
	Eosinophilic microabscesses	4	5	0.44
	Lamina propria fibrosis	4^#^	5	1
**PPI therapy**	PPI dose (mg/kg/d ± SEM)	1.1±0.2	0.9±0.1	0.99
	PPI duration (months ± SEM)	4.6±0.9	2.9±0.3	0.91

*Comparison between PPI responders and PPI non-responders using t-tests or Fischer's exact test. ^#^4 of 5 cases had sufficient lamina propria to evaluate for fibrosis.

For the total 10 study patients, the mean age was 11 years (range 4–16 years), 8 were male, and all were Caucasian. Five patients had a history of atopy (2 allergic rhinitis, 1 eczema, 1 asthma alone, 1 asthma and food allergies). Presenting symptoms included dysphagia (50%), weight loss/poor weight gain (40%), vomiting (40%), abdominal pain (30%), oral aversion (10%), and food impaction (10%). The index endoscopy demonstrated furrows (80%), rings (30%), strictures (10%), white plaques (50%), and edema (50%). Histopathological features included basal cell hyperplasia (100%), spongiosis (100%), papillary elongation (100%), and eosinophilic microabscesses (90%). Nine patients had subepithelial lamina propria in their biopsy specimens, and fibrosis was seen in all 9; one patient did not have sufficient subepithelial lamina propria for evaluation of subepithelial fibrosis, but fibrosis was seen in the lamina propria present in the rete papillae.

### Pre- and Post-PPI Treatment Peak Eosinophil Counts and Histological Features

**Figure 2:**
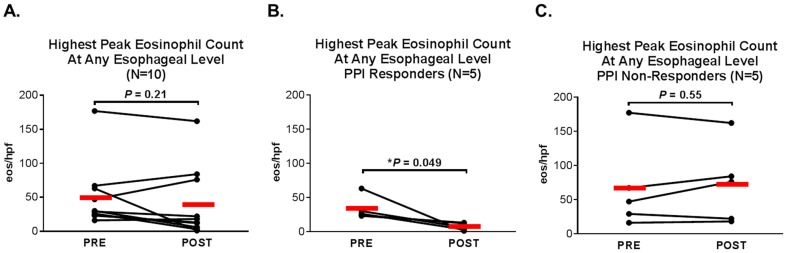
Highest peak eosinophil count at any esophageal level. Highest pre- and post-PPI treatment peak eosinophil counts in (A) all PPI-treated cases, (B) PPI responders, and (C) PPI non-responders. Red bars represent means.

The mean of the highest peak eosinophil count recorded at any level in the esophagus (proximal, mid or distal) for the total 10 patients did not decrease significantly with PPI treatment (Figure 2A). However, the mean of the highest peak eosinophil count fell significantly (from 33 to 7 eos/hpf, P = 0.049) (Figure 2B) in the PPI responders, and did not change significantly in the PPI non-responders (Figure 2C).

Pre- and post-treatment biopsies from all three levels of the esophagus were available for most, but not all of the 10 patients. After PPI treatment, the mean peak eosinophil count decreased, but not significantly, in the proximal esophagus (from 40 to 16 eos/hpf, P = 0.28), changed little in the mid esophagus (30 vs. 27 eos/hpf, P = 0.80) and increased, but not significantly, in the distal esophagus (from 26 to 42 eos/hpf, P = 0.41) (Figure S1).

Histological findings of basal cell hyperplasia, spongiosis, and papillary elongation were seen in all 10 cases prior to PPI treatment. These three features resolved in 2 of the 5 PPI responders, but in none of the non-responders (Table S1). Nine patients (4 PPI responders, 5 PPI non-responders) had eosinophilic microabscesses at baseline, and PPI treatment resulted in complete resolution of microabscesses in all of the 4 PPI responders and in 2 of the 5 PPI non-responders.

### Pre- and Post-PPI Treatment Eotaxin-3-Positive Esophageal Epithelial Cells

**Figure 3:**
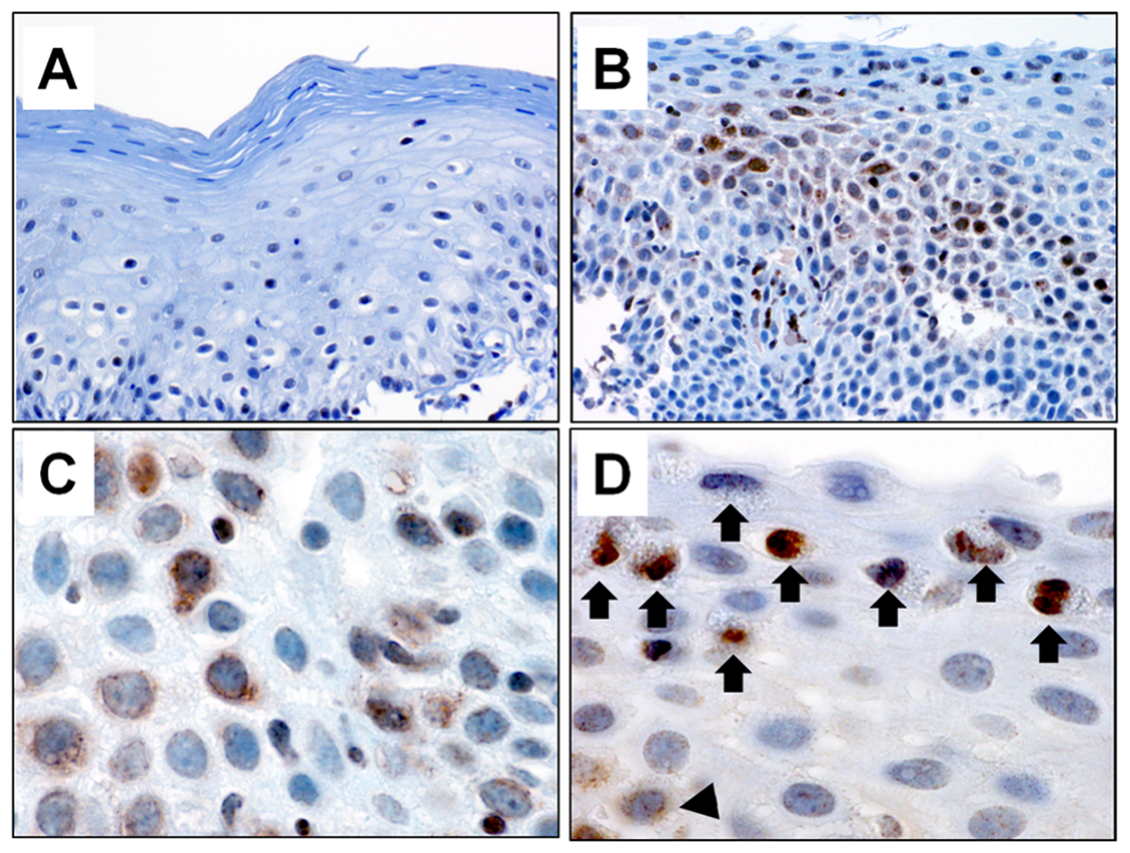
Eotaxin-3 immunostaining of esophageal biopsy specimens. (A) Normal squamous epithelium shows no eotaxin-3 immunostaining. (B) Eotaxin-3 labels epithelial cells with variable intensity in biopsies with esophageal eosinophilia. (C) High power magnification (1000x) identifies a granular cytoplasmic staining that is perinuclear. (D) Eosinophils also showed eotaxin-3 immunostaining, but were readily identified by their morphological features and excluded from quantification. Arrows indicate eosinophils with eotaxin-3 labeling. The isolated arrowhead in the bottom left indicates a squamous epithelial cell with weak perinuclear labeling.

We found variable intensities of eotaxin-3 immunostaining of squamous epithelial cells within and between biopsy specimens from patients with esophageal eosinophilia (Figure 3B); no eotaxin-3 labeling was noted in squamous epithelial cells from a normal control esophagus (Figure 3A). High power magnification (1000x) revealed a granular, perinuclear pattern of cytoplasmic staining in the squamous cells (Figure 3C). Eosinophils also demonstrated immunostaining by eotaxin-3, but eosinophils were readily identified by their distinctive morphology and were excluded from quantification (Figure 3D).

**Figure 4:**
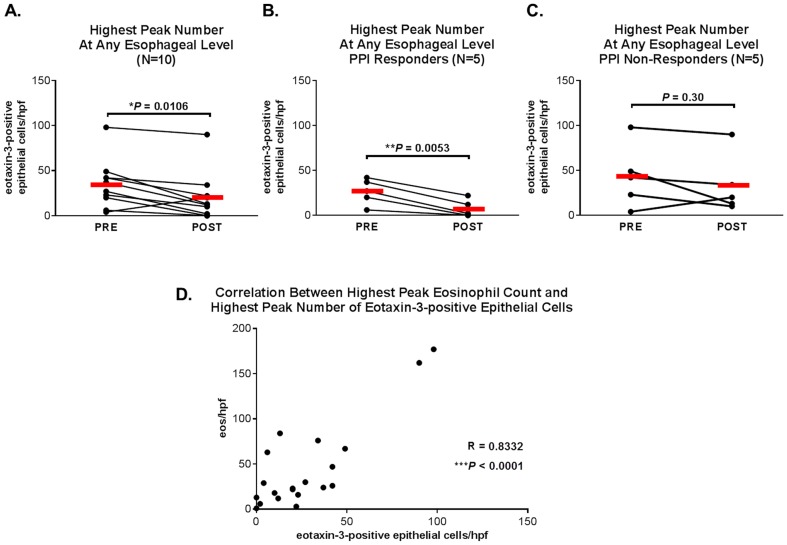
Highest peak number of eotaxin-3-positive epithelial cells at any esophageal level and correlation with highest peak eosinophil count. Highest pre- and post-PPI treatment peak number of eotaxin-3-positive epithelial cells in (A) all PPI-treated cases, (B) PPI responders, and (C) PPI non-responders. Red bars represent means. (D) Correlation between highest peak eosinophil count and highest peak number of eotaxin-3-positive epithelial cells.

The mean of the highest peak number of eotaxin-3-positive squamous epithelial cells at any esophageal level (proximal, mid or distal) for the total 10 patients decreased significantly with PPI treatment (from 35 to 20 cells/hpf, P = 0.0106) (Figure 4A). The mean of the highest peak number of eotaxin-3-positive cells fell significantly in the PPI responders (from 26 to 7 cells/hpf, P = 0.0053) (Figure 4B), and decreased, but not significantly, in the PPI non-responders (Figure 4C). There was a strong correlation between the highest peak eosinophil count and the highest peak number of eotaxin-3-positive epithelial cells (R = 0.8332, P<0.0001) (Figure 4D).

**Figure 5:**
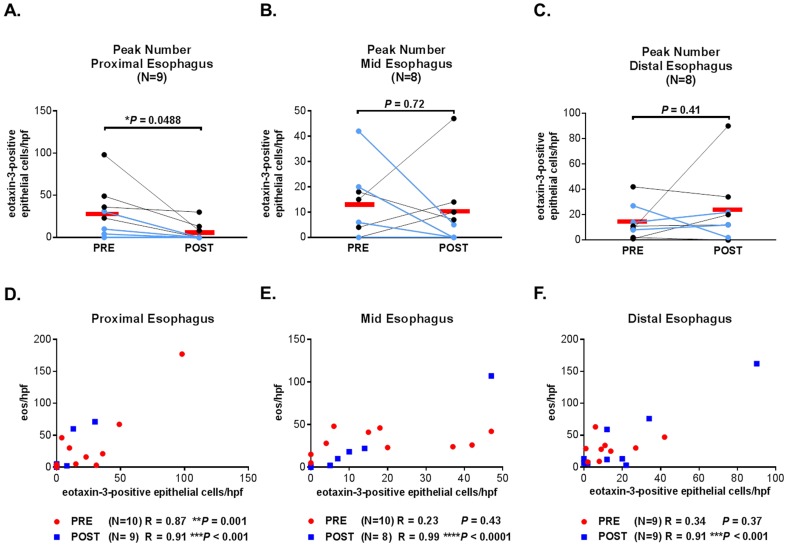
Peak number of eotaxi-3-positive epithelial cells at each esophageal level and correlation with peak eosinophil count. Pre- and post-PPI treatment peak number of eotaxin-3-positive epithelial cells in the (A) proximal, (B) mid, and (C) distal esophagus. Light blue lines represent PPI Responders. Black lines represent PPI Non-responders. Red bars represent means. Correlations between peak eosinophil count and peak number of eotaxin-3-positive epithelial cells are shown pre- and post-PPI treatment in the (D) proximal, (E) mid, and (F) distal esophagus.

Pre- and post-treatment biopsies from all three levels of the esophagus were available for most, but not all of the 10 patients. Interestingly, after PPI treatment, the mean peak number of eotaxin-3-positive epithelial cells decreased significantly in the proximal esophagus (from 28 to 6 cells/hpf, P = 0.049) (Figure 5A), decreased minimally in the mid esophagus (from 13 to10 cells/hpf, P = 0.72) (figure 5B), and increased, but not significantly, in the distal esophagus (from 14 to 24 cells/hpf, P = 0.41) (Figure 5C). In the proximal esophagus, the peak number of eotaxin-3-positive epithelial cells correlated strongly with the peak eosinophil count, both before and after treatment (Figure 5D). In the mid and distal esophagus, there were strong correlations between the peak number of eotaxin-3-positive epithelial cells and peak eosinophil count after treatment, but not before treatment (Figure 5E-F).

### Acid and Bile Salt Effects and PPI Anti-Inflammatory Effects on IL-13-Induced Eotaxin-3 Protein Secretion by Esophageal Epithelial Cells

Although PPI effects in reducing acid reflux are strongest in the *distal* esophagus, we found that PPIs decreased eotaxin-3 expression significantly only in the *proximal* esophagus. This suggested that acidic refluxate might influence eotaxin-3 expression by esophageal epithelial cells. To explore this possibility, we studied the effects of simulated gastroesophageal refluxate solutions (containing acid and/or bile salts) on eotaxin-3 secretion by esophageal epithelial cells with and without IL-13 stimulation.

**Figure 6:**
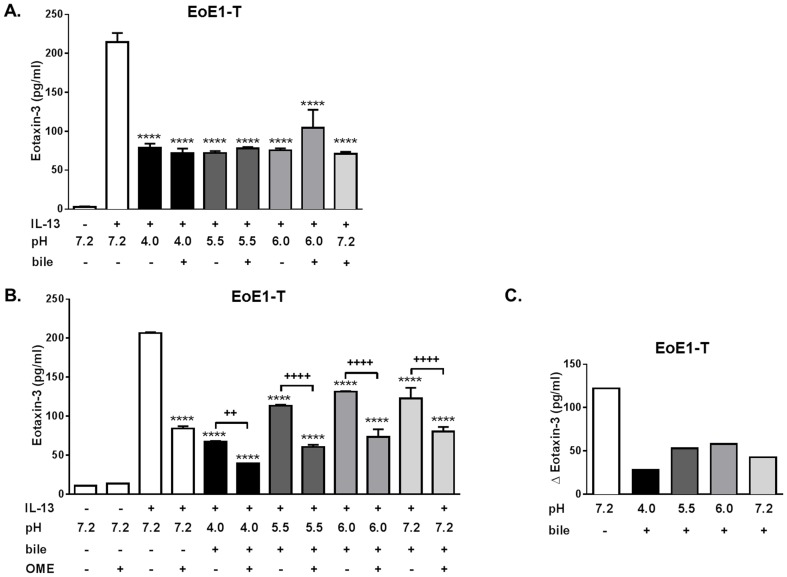
Acid and bile salt effects and PPI anti-inflammatory effects on IL-13-induced eotaxin-3 protein secretion in EoE1-T cells. (A) Acid and/or bile salt effects on IL-13-induced eotaxin-3 protein secretion. Note the profound suppression of eotaxin-3 protein secretion with exposures to acid alone, bile salts alone, or the combination of acid and bile salts. Data are mean ± SEM of 2 experiments. ****P<0.0001 compared to IL-13 alone (pH 7.2 with no bile). (B) Combined effects of acid-bile salt medium and omeprazole (OME) on IL-13-induced eotaxin-3 protein secretion. Data are mean ± SEM of 2 experiments. ****P<0.0001 compared to IL-13 alone (pH 7.2, no bile, no OME). ^++^P<0.01and ^++++^P<0.0001. (C) Graph depicts the magnitude of the decrease in IL-13-stimulated eotaxin-3 secretion achieved by omeprazole for each acid-bile salt exposure condition.

**Figure 7:**
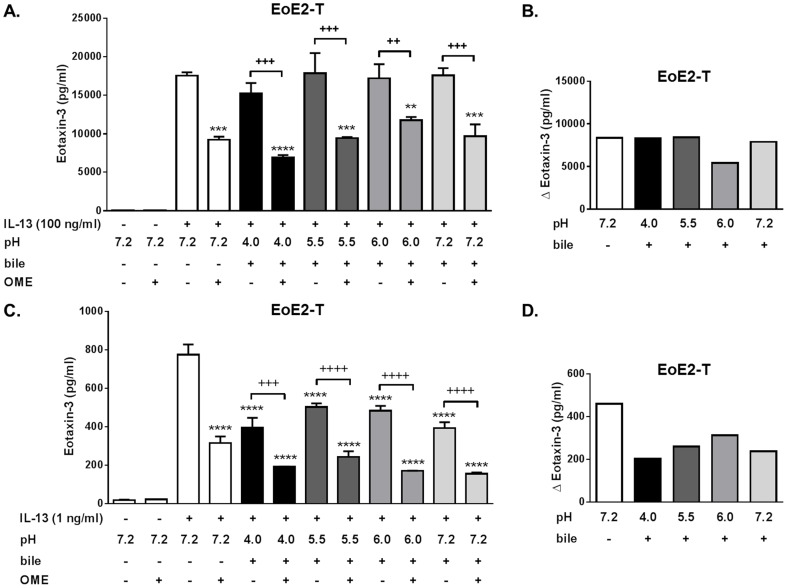
Acid and bile salt effects and PPI anti-inflammatory effects on IL-13-induced eotaxin-3 protein secretion in EoE2-T cells. (A) Combined effects of acid-bile salt medium and omeprazole (OME) on eotaxin-3 protein secretion in EoE2-T cells stimulated with 100 ng/ml of IL-13. Data are mean ± SEM of 2 experiments. **P<0.01, ***P<0.001, and ****P<0.0001 compared to IL-13 alone (pH 7.2, no bile, no OME). ^++^P<0.01 and ^+++^P<0.001. (B) Graph depicts the magnitude of the decrease in IL-13-stimulated eotaxin-3 secretion achieved by omeprazole for each acid-bile salt exposure condition in Figure 7A. (C) Combined effects of acid-bile salt medium and omeprazole on eotaxin-3 protein secretion in EoE2-T cells stimulated with 1 ng/ml of IL-13. Data are mean ± SEM of 2 experiments. ****P<0.0001 compared to IL-13 alone (pH 7.2, no bile, no OME). ^++^P<0.01 and ^+++^P<0.001. (D) Graph depicts the magnitude of the decrease in IL-13-stimulated eotaxin-3 secretion achieved by omeprazole for each acid-bile salt exposure condition in Figure 7C.

**Figure 8:**
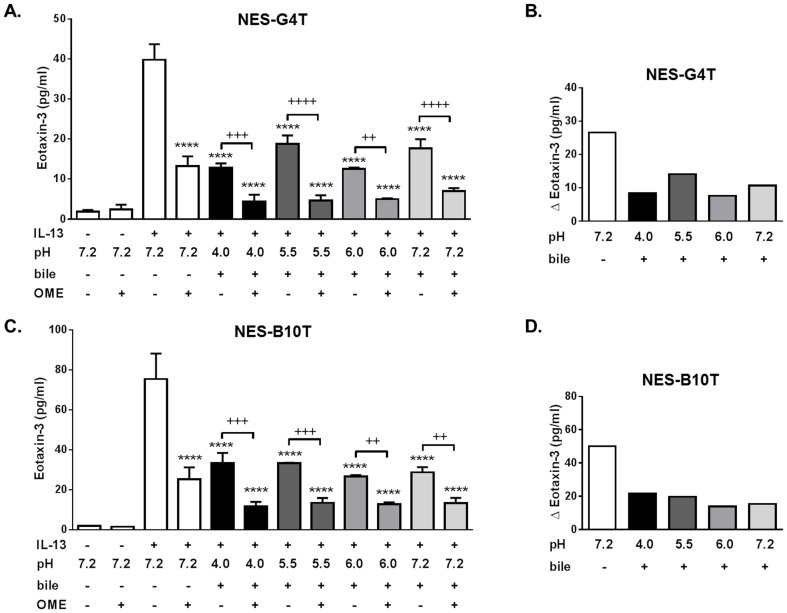
Acid and bile salt effects and PPI anti-inflammatory effects on IL-13-induced eotaxin-3 protein secretion in GERD cells. (A) Combined effects of acid-bile salt medium and omeprazole (OME) on IL-13-induced eotaxin-3 protein secretion in NES-G4T. Data are mean ± SEM of 2 experiments. ****P<0.0001 compared to IL-13 alone (pH 7.2, no bile, no OME). ^++^P<0.01, ^+++^P<0.001, ^++++^P<0.0001. (B) Graph depicts the magnitude of the decrease in IL-13-stimulated eotaxin-3 secretion achieved by omeprazole for each acid-bile salt exposure condition in NES-G4T. (C) Combined effects of acid-bile salt medium and omeprazole on IL-13-induced eotaxin-3 protein secretion in NES-B10T. Data are mean ± SEM of 2 experiments. ****P<0.0001 compared to IL-13 alone (pH 7.2, no bile, no OME). ^++^P<0.01 and ^+++^P<0.001. (D) Graph depicts the magnitude of the decrease in IL-13-stimulated eotaxin-3 secretion achieved by omeprazole for each acid-bile salt exposure condition in NES-B10T.

Without IL-13 stimulation, we found little basal secretion of eotaxin-3 by esophageal epithelial cells (Figures 6A, 7A, 8A). In EoE1-T cells, this low basal level of eotaxin-3 protein secretion was not affected significantly by exposure to acid and/or bile salts (Figure S2). Next, we explored the effects of acid and bile salts on IL-13-stimulated eotaxin-3 secretion by EoE1-T cells. As seen in our earlier studies, IL-13 (100 ng/ml) added to neutral control medium induced a significant and profound increase in eotaxin-3 protein secretion (Figure 6A, white bar) [5], [13]. This IL-13 stimulated increase in eotaxin-3 secretion was attenuated significantly by exposure to acid alone (pH levels of 4.0 to 6.0), bile salts alone, or the combination of acid and bile salts) (Figure 6A, black and gray bars). We found no significant differences between acid alone, bile salts alone, and the combination (at each pH level studied) in suppressing IL-13-stimulated eotaxin-3 secretion. Therefore, we selected to use the combination of acid and bile salts, a more physiological representation of typical gastroesophageal reflux episodes, for subsequent experiments.

In earlier studies, we showed that PPIs block Th2-cytokine-stimulated eotaxin-3 secretion through mechanisms independent of effects on gastric acid secretion (i.e. acid-independent, anti-inflammatory PPI effects) [5], [13]. Now, we studied how acid and bile salts affect omeprazole's ability to block IL-13-stimulated eotaxin-3 secretion by EoE1-T cells. As in our earlier studies, omeprazole significantly suppressed IL-13-stimulated eotaxin-3 secretion at neutral conditions without bile salts (Figure 6B) [5], [13]. As in the experiments shown in Figure 6A, exposure to acid-bile salt medium also significantly suppressed IL-13-stimulated eotaxin-3 secretion (Figure 6B). Omeprazole further suppressed eotaxin-3 secretion at each acid-bile salt exposure condition, but not as profoundly as at neutral conditions without bile salts (Figures 6B and C).

We repeated these experiments in EoE2-T cells. In response to IL-13 in a dose of 100 ng/ml, however, the amount of eotaxin-3 protein secreted by EoE2-T cells is almost two orders of magnitude greater than that secreted by EoE1-T cells (Figure 7A). Unlike EoE1-T cells, acid and bile salts did not significantly affect eotaxin-3 secretion by EoE2-T cells stimulated with100 ng/ml of IL-13 (Figure 7A). Also unlike EoE1-T cells, omeprazole suppressed that IL-13-stimulated eotaxin-3 secretion in EoE2-T cells exposed to acid and bile salts to a level comparable to that achieved at neutral control conditions (Figure 7B). In separate experiments, we confirmed that neither acid alone, bile salts alone, nor the combination suppressed the robust secretion of eotaxin-3 stimulated by IL-13 in a dose of 100 ng/ml (Figure S3). Therefore, we repeated our experiments using a lower dose of IL-13 (1 ng/ml) at which EoE2-T cells secrete eotaxin-3 in amounts comparable to EoE1-T cells. With this lower dose of IL-13, results in EoE2-T cells were similar to those in EoE1-T cells. Specifically, all acid-bile salt exposure conditions significantly suppressed IL-13-induced eotaxin-3 secretion (Figure 7C), and the magnitude of omeprazole-induced suppression in cells exposed to acid and bile salts was less than that at neutral conditions without bile salts (Figure 7D).

Finally, to determine whether these findings are specific to esophageal cells from patients with EoE, we examined acid-bile salt effects on IL-13-stimulated eotaxin-3 secretion in cell lines derived from patients with GERD (NES-G4T and NES-B10T). We used IL-13 in a dose of 100 ng/ml, since NES-G4T and NES-B10T have previously demonstrated lower ranges of Th2 cytokine-stimulated eotaxin-3 secretion [5]. In both GERD cell lines, acid-bile salt exposures significantly suppressed IL-13-induced-eotaxin-3 secretion (Figure 8A&C). The addition of omeprazole further suppressed eotaxin-3 secretion but, as in the EoE cell lines, the magnitude of that suppression was considerably less than that at neutral conditions without bile salts (Figure 8B&D).

## Discussion

In esophageal biopsy specimens from children with esophageal eosinophilia, we have demonstrated a strong correlation between the peak number of eosinophils and the peak number of eotaxin-3-immunostained epithelial cells. Although there may well be other factors contributing to esophageal eosinophilia, this strong correlation suggests that eotaxin-3 produced by epithelial cells plays a major role in attracting eosinophils to the esophagus. To our surprise, we found that PPI treatment significantly decreased eotaxin-3 expression (which correlated with eosinophil count) only in the *proximal* esophagus, even though PPI effects in reducing acid reflux are strongest in the *distal* esophagus. To explore possible mechanisms for this disparity, we studied acid and bile salt effects on IL-13-stimulated eotaxin-3 secretion in esophageal squamous cell lines, and found that exposures to acid alone, bile salts alone, and the combination of acid and bile salts have suppressive effects on IL-13-induced eotaxin-3 secretion. Acid-independent, anti-inflammatory effects of omeprazole seem to further suppress that eotaxin-3 secretion, but the magnitude of that suppression is attenuated under acid-bile salt exposure conditions.

**Figure 9:**
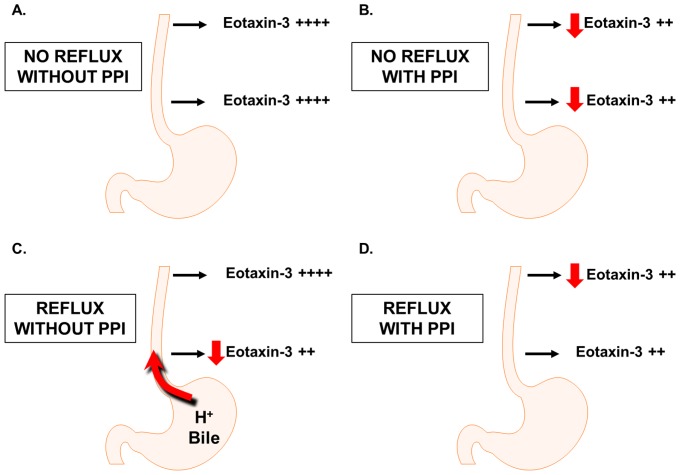
Conceptual summary of *in vitro* findings. (A) In patients with Th2-cytokine driven esophageal eosinophilia who have little or no reflux, epithelial cell expression of eotaxin-3 will be high in both the proximal and distal esophagus. (B) When patients without reflux are given PPIs, epithelial cells in both the proximal and distal esophagus can respond to the acid-independent, anti-inflammatory effects, which decrease eotaxin-3 expression in both locations. (C) For patients who have Th2-cytokine driven esophageal eosinophilia and gastroesophageal reflux, eotaxin-3 expression in the distal esophagus might be suppressed by reflux, while eotaxin-3 expression remains high in the proximal esophagus where reflux exposure is minimal. (D) When patients with reflux are treated with PPIs, the proximal esophagus responds to the acid-independent, anti-inflammatory PPI effects in suppressing eotaxin-3. In the distal esophagus, however, refluxed acid already might be suppressing eotaxin-3 expression. PPI antisecretory effects could increase cytokine-stimulated eotaxin-3 expression by decreasing acid reflux, while PPI anti-inflammatory effects could decrease eotaxin-3 expression. Thus, PPI treatment might have little net effect on eotaxin-3 expression in the distal esophagus.

Our *in vitro* observations provide a possible explanation for the disparate PPI effects that we observed on eotaxin-3 expression in the proximal and distal esophagus of our patients with esophageal eosinophilia. In patients with Th2-cytokine driven esophageal eosinophilia who have little or no reflux, epithelial cell expression of eotaxin-3 will be high in both the proximal and distal esophagus (Figure 9A). When those patients are given PPIs, epithelial cells in both the proximal and distal esophagus can respond to the acid-independent, anti-inflammatory effects of the PPI, which profoundly diminish eotaxin-3 expression in both locations (Figure 9B). For patients who have Th2-cytokine driven esophageal eosinophilia and gastroesophageal reflux, in contrast, eotaxin-3 expression in the distal esophagus might be suppressed by reflux, while eotaxin-3 expression remains high in the proximal esophagus where reflux exposure is minimal (Figure 9C). When those patients are treated with PPIs, the proximal esophagus responds to the acid-independent, anti-inflammatory PPI effects in suppressing eotaxin-3, and the decreased expression of this eosinophil chemoattractant results in a commensurate reduction in proximal esophageal eosinophilia (Figure 9D). In the distal esophagus, however, refluxed acid already might be suppressing eotaxin-3 expression (Figure 9C). By decreasing that acid reflux, PPI treatment will free those distal esophageal cells from acid-induced suppression of eotaxin-3. Although the anti-inflammatory effects of PPIs might decrease eotaxin-3 expression, those effects would be masked in the distal esophagus by the increase in eotaxin-3 expression that results from the suppression of acid reflux. Thus, PPI treatment might have little net effect on eotaxin-3 expression in the distal esophagus.

Even patients who have esophageal eosinophilia without clinically apparent GERD might have disparate PPI effects on the proximal and distal esophagus. Esophageal pH monitoring studies have documented that the distal esophagus can be exposed to acid (pH <4) for 4% to 5% of a 24-hour monitoring period in normal adults, and for 8% to 13% of the monitoring period in normal newborns and infants [20], [21]. In contrast, the proximal esophagus normally is exposed to acid for <1% of the day [22]. In the distal esophagus, conceivably, even the “normal” amount of reflux might be sufficient to suppress eotaxin-3 secretion.

In this and earlier studies, we have observed great variability in the amount of eotaxin-3 secretion induced by Th2 cytokine stimulation in esophageal squamous cells from different patients. In response to IL-13 in a dose of 100 ng/ml, for example, the amount of eotaxin-3 protein secreted by EoE2-T cells is almost two orders of magnitude greater than that secreted by EoE1-T cells. At that high level of cytokine-stimulated eotaxin-3 secretion, we could not demonstrate its suppression by acid and bile salts and, even though omeprazole did suppress eotaxin-3 secretion significantly, the levels of secretion remained profoundly elevated nevertheless. When we repeated our experiments using a lower dose of IL-13 (1 ng/ml) at which EoE2-T cells secrete eotaxin-3 in amounts comparable to EoE1-T cells, the two cell lines responded similarly to acid, bile salts and omeprazole. These findings suggest that, for patients with very high cytokine-stimulated levels of eotaxin-3 secretion, the effects of acid, bile and PPIs might not be clinically apparent. Individual differences in esophageal sensitivity to Th2 cytokine-stimulated eotaxin-3 secretion might explain why some patients respond to PPI treatment and others do not.

In a study published in abstract form, Molina-Infante *et al*. observed that PPI treatment caused transcriptional downregulation of eotaxin-3 mRNA in *both* the proximal and distal esophagus of adults with PPI-REE [23]. Unlike our study, which used immunohistochemical staining to demonstrate the epithelial cells responsible for eotaxin-3 protein production, those investigators evaluated eotaxin-3 mRNA levels in esophageal biopsy specimens containing numerous non-epithelial cells, including eosinophils, which also can express eotaxin-3. Since, by definition, eosinophils decrease with PPI treatment in PPI-REE, it is possible that the observed reductions in post-treatment eotaxin-3 mRNA levels might have been the result rather than the cause of the decreased eosinophils, and may not reflect what is occurring in the epithelial cells. It is also possible that acid and bile might affect the translation or post-translational degradation of eotaxin-3 rather than its transcription. Further studies are needed to establish the mechanisms responsible for acid, bile salt, and PPI effects on eotaxin-3 expression.

For our experiments delineating the effects of pH and bile on eotaxin-3 secretion, we exposed esophageal squamous cells to a medium containing a mixture of acid and bile salts simulating the composition of gastric refluxate described in patients with GERD [18]–[20]. We felt that exposure to acid-bile salt medium at various pH levels would provide more physiologically relevant data on the effects of acidic reflux than exposure to various strengths of hydrochloric acid alone. For esophageal pH monitoring studies, acid reflux typically is defined by an esophageal pH drop below 4. However, weakly acidic gastroesophageal reflux (with pH levels between 4 and 7) has been shown to occur frequently in patients with GERD who take PPIs [24]. Therefore, we exposed our cells to pH levels ranging from 4.0 to 7.2 to simulate the effects of gastroesophageal reflux in patients on and off PPIs. We did not expose esophageal squamous cells to medium with pH levels <4, because such strongly acidic medium kills these cells in culture.

We identified 264 children with esophageal eosinophilia who were treated at our medical center from 2008 to 2012, but we found only 10 who qualified for our study. There are several reasons for the large number of patient exclusions. Endoscopy in pediatric patients usually involves general anesthesia with its attendant inconvenience, expense and risks, including recent concerns regarding neurotoxic effects of anesthesia on the developing brain [25]. Most of our patients with esophageal eosinophilia were seen prior to publication of the 2011 updated consensus statement recognizing the entity of PPI-REE [6]. Therefore, our children evaluated for EoE-like symptoms usually were given an empiric trial of PPIs (without a prior endoscopy) to exclude GERD, as recommended in the 2007 consensus guidelines [15]. Patients whose symptoms resolved on PPIs usually were assumed to have GERD, and were continued on PPIs without endoscopic evaluation. Therefore, the large majority of our index endoscopies showing esophageal eosinophilia were performed for patients whose symptoms had not responded to PPIs. Since these patients had already received PPIs, they were not eligible for our study. Thus, we could identify only 40 potential subjects who had esophageal biopsies taken both before and after PPI treatment, and we excluded 30 of those because they had received other, concomitant treatments during their PPI therapy (steroids and/or diet). Consequently, in clinical practice it is difficult to find pediatric patients with esophageal eosinophilia who have had endoscopic examinations before and after a course of PPI therapy alone.

Five (50%) of our 10 study patients with esophageal eosinophilia had PPI-REE, a frequency within the range reported by others [26]–[31]. Compared to the PPI non-responders, our patients with PPI-REE had more frequent symptoms of weight loss/poor weight gain and vomiting, and their baseline peak eosinophil counts were somewhat lower. No other clinical features distinguished our PPI responders from non-responders. Our patient numbers are too small for meaningful multivariable analysis, but it seems unlikely that the minor clinical differences between the groups that we observed would have clinical utility for identifying patients with esophageal eosinophilia who are likely to respond to PPIs. Thus, our findings in children are similar to those in adults with esophageal eosinophilia reported by Dellon *et al*., who found that no clinical or endoscopic features independently distinguished PPI responders from non-responders. [32]

Our study has a number of limitations in addition to its small sample size. It suffers from limitations inherent in a retrospective investigation conducted at a single medical center. We cannot assess for patient compliance with PPI therapy, for concomitant use of unprescribed therapies, and for seasonal and environmental influences. Also, our patients did not have esophageal pH-impedance monitoring studies. With our findings that differences in acid exposure might underlie the different effects of PPIs on eotaxin-3 expression in the proximal and distal esophagus, it will be important for future studies to measure the pH, frequency, duration and proximal extent of gastroesophageal reflux to elucidate the role of acid exposure in modifying PPI-responsiveness.

In conclusion, in this study designed to explore the contributions of the acid-inhibitory and the acid-independent, anti-inflammatory effects of PPIs on esophageal eosinophilia, we have found that PPIs decrease eotaxin-3 expression significantly in the proximal but not the distal esophagus. This finding was contrary to our expectation that PPI effects in reducing acid reflux would preferentially benefit the distal esophagus. Our *in vitro* finding that exposure to acid and bile inhibits Th2-cytokine-stimulated eotaxin-3 secretion by esophageal epithelial cells might account for the surprising results. In the distal esophagus, exposure to acid reflux might suppress eotaxin-3 expression, rendering it less sensitive to the acid-independent, anti-inflammatory effect of PPIs. In the proximal esophagus, those acid-independent, anti-inflammatory suppressive effects of PPIs on eotaxin-3 are manifest most prominently. Further studies are needed to substantiate and expand upon our findings. However, since it appears that PPIs can have disparate effects on eotaxin-3 expression in the proximal and distal esophagus, clinicians should consider taking biopsy samples from both the proximal and distal esophagus when assessing the effects of PPIs on esophageal eosinophilia.

## Supporting Information

Figure S1 Pre- and post-PPI treatment peak eosinophil counts at each esophageal level. (A) Proximal, (B) mid, and (C) distal esophagus. Red bars represent means.
(PDF)

Figure S2 Acid and bile salt effects on basal eotaxin-3 protein secretion (without IL-13 stimulation) in EoE1-T. NS, not significant compared to neutral control media (pH 7.2 with no bile).
(PDF)

Figure S3 Acid alone, bile alone, and the combination had no suppressive effect on EoE2-T cells stimulated with IL-13 (100 ng/ml). NS, not significant compared to pH-neutral control media (IL-13, pH 7.2, no bile).
(PDF)

Table S1 Pre- and Post-PPI Treatment Histological Findings.
(DOCX)
