# Supplementary material for: Proton Pump Inhibitors Decrease Eotaxin-3 Expression in the Proximal Esophagus of Children with Esophageal Eosinophilia
Source: PLoS One. 2014 Jul 2;9(7):e101391. doi: 10.1371/journal.pone.0101391 (PMC4079672; doi:10.1371/journal.pone.0101391)
Supplement: Figure S1 — Pre- and post-PPI treatment peak eosinophil counts at each esophageal level. (A) Proximal, (B) mid, and (C) distal esophagus. Red bars represent means. (PDF) [file pone.0101391.s001.pdf]

Figure S1

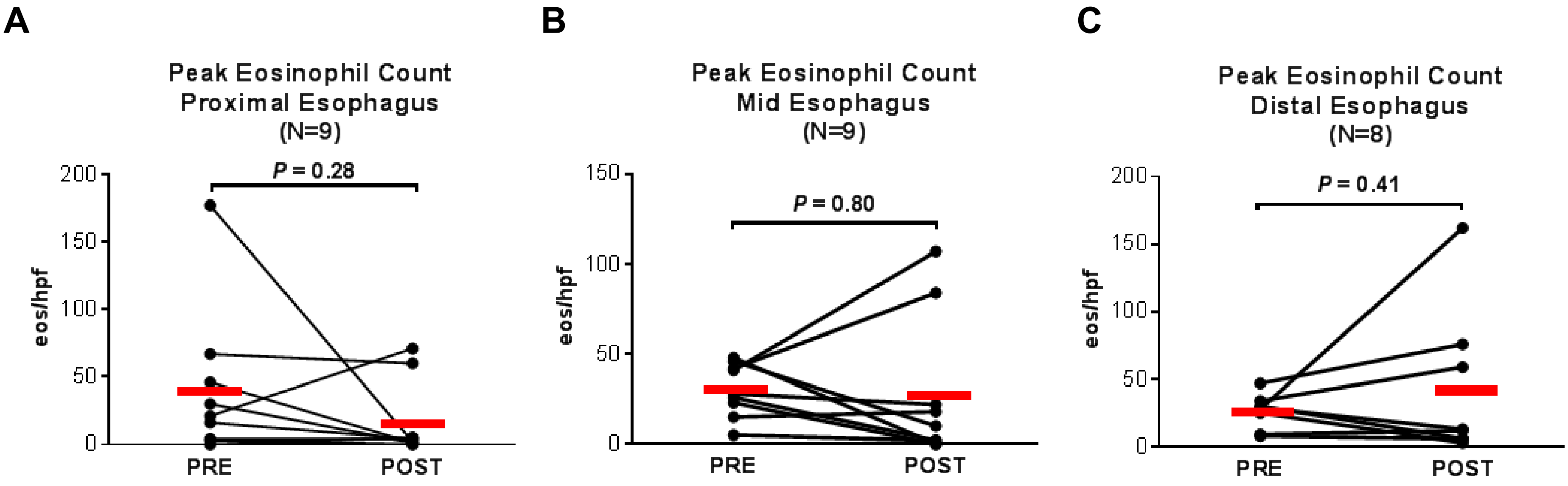

Pre- and post-PPI treatment peak eosinophil counts in (A) proximal, (B) mid, and (C) distal esophagus. Red bars represent means.
