# Supplementary material for: Proton Pump Inhibitors Decrease Eotaxin-3 Expression in the Proximal Esophagus of Children with Esophageal Eosinophilia
Source: PLoS One. 2014 Jul 2;9(7):e101391. doi: 10.1371/journal.pone.0101391 (PMC4079672; doi:10.1371/journal.pone.0101391)
Supplement: Figure S2 — Acid and bile salt effects on basal eotaxin-3 protein secretion (without IL-13 stimulation) in EoE1-T. NS, not significant compared to neutral control media (pH 7.2 with no bile). (PDF) [file pone.0101391.s002.pdf]

Figure S2

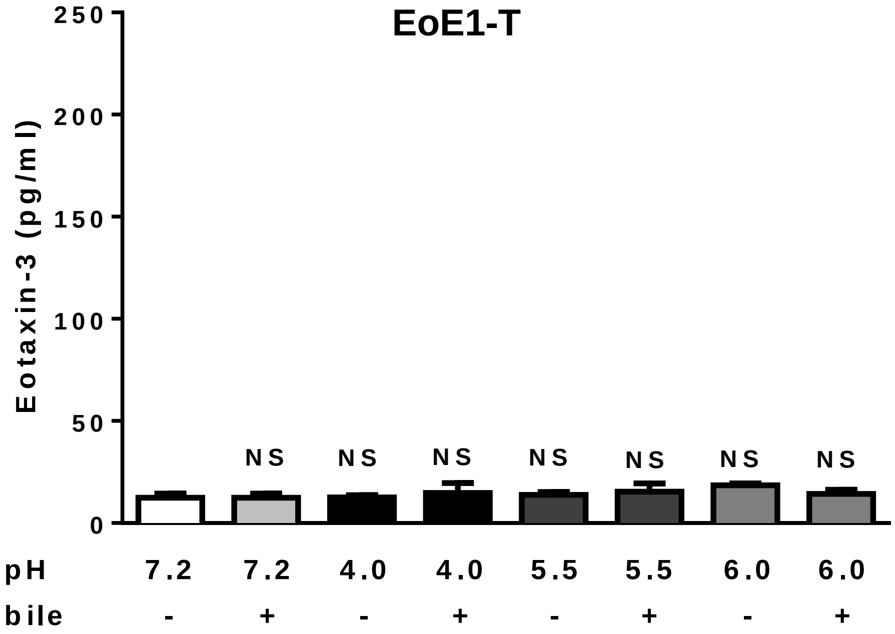

Acid and bile salt effects on basal eotaxin-3 protein secretion (without IL-13 stimulation) in EoE1-T. NS, not significant compared to neutral control media (pH 7.2 with no bile).
