# Supplementary material for: Proton Pump Inhibitors Decrease Eotaxin-3 Expression in the Proximal Esophagus of Children with Esophageal Eosinophilia
Source: PLoS One. 2014 Jul 2;9(7):e101391. doi: 10.1371/journal.pone.0101391 (PMC4079672; doi:10.1371/journal.pone.0101391)
Supplement: Figure S3 — Acid alone, bile alone, and the combination had no suppressive effect on EoE2-T cells stimulated with IL-13 (100 ng/ml). NS, not significant compared to pH-neutral control media (IL-13, pH 7.2, no bile). (PDF) [file pone.0101391.s003.pdf]

Figure S3

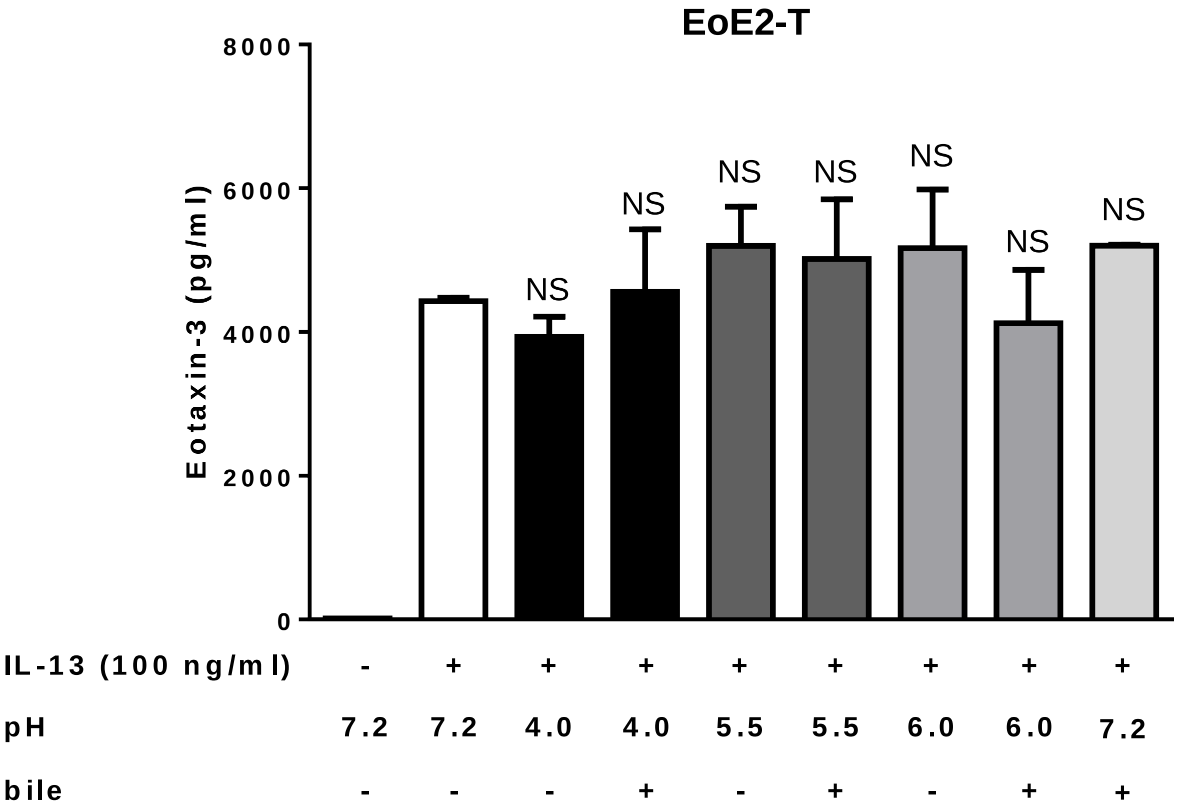

Acid alone, bile alone, and the combination had no suppressive effect on EoE2-T cells stimulated with IL-13 (100 ng/ml). NS, not significant compared to pH-neutral control media (IL-13, pH 7.2, no bile).
