# Supplementary material for: Proton Pump Inhibitors Decrease Eotaxin-3 Expression in the Proximal Esophagus of Children with Esophageal Eosinophilia
Source: PLoS One. 2014 Jul 2;9(7):e101391. doi: 10.1371/journal.pone.0101391 (PMC4079672; doi:10.1371/journal.pone.0101391)
Supplement: Table S1 — Pre- and Post-PPI Treatment Histological Findings. (DOCX) [file pone.0101391.s004.docx]

**Table S1. Pre- and Post-PPI Treatment Histological Findings**

|  | **PPI Responders (N=5)** | | **PPI Non-Responders (N=5)** | |
| --- | --- | --- | --- | --- |
|  | **PRE** | **POST** | **PRE** | **POST** |
| **Basal Hyperplasia** | 5 (100%) | 3 (60%) | 5 (100%) | 5 (100%) |
| **Spongiosis** | 5 (100%) | 3 (60%) | 5 (100%) | 5 (100%) |
| **Papillary Elongation** | 5 (100%) | 3 (60%) | 5 (100%) | 5 (100%) |
| **Eosinophilic Microabscesses** | 4 (80%) | 0 (0%) | 5 (100%) | 3 (60%) |
